# Dissociations of oral foci of infections with infectious complications and survival after haematopoietic stem cell transplantation

**DOI:** 10.1371/journal.pone.0225099

**Published:** 2019-12-18

**Authors:** Matti Mauramo, Patricia Grolimund, Adrian Egli, Jakob Passweg, Jörg Halter, Tuomas Waltimo

**Affiliations:** 1 Department of Oral and Maxillofacial Diseases, University of Helsinki and Helsinki University Hospital, Helsinki, Finland; 2 Department of Pathology, Haartman Institute and HUSLab, Helsinki University Central Hospital, Helsinki, Finland; 3 Department for Oral Health & Medicine, UZB University Centre for Dental Medicine Basel, University of Basel, Basel, Switzerland; 4 Division of Clinical Microbiology, University Hospital Basel, Basel, Switzerland; 5 Applied Microbiology Research, Department of Biomedicine, University of Basel, Basel, Switzerland; 6 Department of Haematology, University Hospital Basel, Basel, Switzerland; Shanghai Jiao Tong University School of Medicine, CHINA

## Abstract

**Introduction:**

Haematopoietic stem cell transplantation (HSCT) recipients are at increased risk for severe infections. This study examined the associations of common oral infections with survival and infectious complications in HSCT recipients.

**Materials and methods:**

All autologous and allogeneic HSCT recipients transplanted in the University Hospital of Basel, Switzerland, between 2008 and 2016 and referred to oral infection control pre-HSCT were included in this retrospective case-control study. All patients had a clinical and a panoramic radiological dental examination taken immediately prior to HSCT. Presence of acute or chronic oral foci of infections, decayed, missing or filled tooth index (DMFT) and radiological attachment loss (RAL) were examined. Survival and infections of the subjects were followed up for 6 months post-HSCT.

**Results:**

Altogether 341 allogeneic and 125 autologous HSCT recipients were included in the study. Within 6 months post-HSCT, 47 (14%) of the allogeneic and 4 (3%) of the autologous recipients died. Oral foci of infections (acute or chronic), DMFT or periodontitis pre-HSCT were not associated with survival 6 months post-HSCT. Oral foci of infections were also not associated with hospital treated infectious diseases or blood culture positive bacteremia during the 6 month follow-up period. Untreated oral foci of infections were not associated with survival or severe infectious complications within 6 months post-HSCT.

**Conclusion:**

The results of this study suggest that radical dental interventions to chronic oral infections could be postponed until post-HSCT.

## Introduction

Haematopoietic stem cell transplantation (HSCT) is a widely used potentially curative treatment for severe haematological diseases including various types of haemato-oncologic diseases. During HSCT, high-dose chemotherapy with or without total body irradiation is utilised to eradicate malignant cells and to produce myelosuppression and immunosuppression [1]. Due to improvements in the transplantation procedures, the number of long-term survivors is constantly increasing [2]. Nevertheless, HSCT remains associated with considerable acute and long-term morbidities, such as graft-versus-host disease (GvHD), organ dysfunction and secondary malignancies. Due to increased survival rates, less severe symptoms are common and gaining more clinical importance. [3–5].

There is increasing evidence that HSCT can cause distressing acute and long-term side effects in the oral cavity, affecting the general wellbeing and quality of life of the patients [5–11]. Previous studies have mostly focused on symptoms related to GvHD, including mucositis and hyposalivation. However, only a few studies have examined the prevalence and consequences of common oral infections in HSCT recipients. Consequently, the existing protocols used for managing oral diseases and associated infectious complications are, hitherto, based on extrapolations from clinical studies and experience among other oncologic diseases or their treatment protocols. These include head and neck cancer and solid organ transplantations [12]. Such extrapolations have revealed dental and oral medicine treatment protocols that differ remarkably between institutions worldwide [13].

In HSCT, strong immunosuppression is mandatory, and radical dental treatment is therefore commonly utilised [14]. This approach has been supported for decades by the National Institute of Health consensus statement on oral complications of cancer therapy (1989), which states that dental foci are potential sources of systemic infections that need to be eliminated or ameliorated before the commencement of an anticancer therapy. Using a decision analysis method, it has been estimated that dental treatment prior to HSCT may reduce systemic infections by 30% [15]. However, evidence on the benefits of dental treatments pre-HSCT is scarce and partly controversial [16]. Thus, a relatively conservative approach, with an emphasis on good oral hygiene and basic oral care, has been advised in a guideline concerning dental care among HSCT patients by the established MASCC/ISOO collaboration [13]. However, much research is needed to provide robust scientific evidence to support the guidelines, and to unify the oral treatment protocols prior, during and after HSCT.

This study examined the associations of common oral infections with survival and infectious complications in HSCT recipients. The hypothesis, based on our clinical experience and studies with comparable populations, was that oral foci of infection do in general not predispose to severe systemic complications post-HSCT, and they can be left untreated pre-HSCT [17].

## Materials and methods

This retrospective study was approved by the Ethikkommission Nordwest- und Zentralschweiz (EKNZ), Switzerland (Ref. Nr. EKNZ:311–10). The study was conducted in the University Hospital of Basel and in the University Center of Dental Medicine Basel, University of Basel, Switzerland. All 446 consecutive adult (>16 years) HSCT recipients transplanted between 1st of January 2008 and 31st of December 2016, and referred to oral infection control pre-HSCT, were included in the study. All data collected from medical records were fully anonymized before accessed. At that time, most patients had already been treated for their underlying disease using standard chemotherapy schemas and had received conditioning chemotherapy either with or without TBI, as previously described [11].

All relevant medical data including stem cell source, total body irradiation, survival and cause of death of the non-survivors were examined. GvHD (GvHD) was graded according to standard grading crite,ria and considered as acute (aGvHD) if the onset of the disease was <100 days post-HSCT. Additionally, infections diagnosed and treated in the hospital within 6 months post-HSCT were examined, and grouped as: 1. any infection, 2. pneumonia, 3. gut infection, or 4. bacterial/fungal/viral-infection. In the course of HSCT, routine blood samples related to the treatment protocol were taken several times, and additional samples also whenever there were signs of infection. All cases of culture positive bacteremia including the bacterial species were collected.

All study subjects had a thorough dental examination by an experienced dentist pre-HSCT in the School of Dental Medicine, University Hospital of Basel, Switzerland. A panoramic dental x-ray was taken from all recipients pre-HSCT and observed by a specialist in radiology giving a written statement. All the dental records, panoramic dental x-rays and associated reports were re-evaluated, with a particular focus on oral foci of infections. Chronic oral foci of infections were inspected from the panoramic dental x-rays and grouped as follows: 1. apical periodontitis, 2. caries profunda with the potential for pulp exposure, 3. furcation involvement, 4. other foci of infection including root remnants, partially erupted wisdom teeth with pericoronitis, initiated endodontic treatment, and cysts. Acute oral foci of infections included: 1. fistula with purulent drainage, 2. symptomatic periapical granuloma, 3. symptomatic deep caries, 4. fresh extraction socket and 5. acute periodontal abscess with purulent drainage. Symptomatic acute oral foci of infections were treated with antibiotics whenever observed prior to the HSCT. To avoid bacteremia in severely ill patients, periodontitis was only examined in terms of radiological attachment level (RAL) from the panoramic dental x-rays. Periodontitis was determined to be present if radiological attachment loss (RAL), e.g. distance between cementoenamel junction (CEJ) and alveolar bone crest, was observed to be > 3mm [18]. The number of caries lesions and the presence of acute or previous carious lesions (decayed, missing or filled tooth (DMFT)-index) were examined with thorough dental examination.

### Statistics

Main outcomes were survival and infectious complications within 6 months post-HSCT. The associations of HSCT related factors, oral foci of infections and the presence of acute or chronic oral diseases with the main outcomes were analysed. Additionally, the associations of oral foci of infections with hospital treated infections and blood culture positive bacteremia were examined. Pearson Chi-square (binary outcomes) and Mann-Whitney U tests (others) were used to determine statistical significance. A p-value of <0.05 was considered as statistically significant. In the models comparing the associations of different groups of chronic oral foci of infections (any chronic oral foci, apical periodontitis, caries profunda, furcation involvement or other foci) with the outcomes, the Bonferroni correction was used and a p-value of 0.01 was considered as statistically significant.

## Results

### Study population

341 allogeneic (mean age at HSCT: 49.4±13.7) and 125 (mean age at HSCT: 55.2±11.3) autologous HSCT recipients were included in the study (Table 1). Pre-HSCT, 25/341 (7%) of the allogeneic recipients had at least one acute oral focus of infection, 167/341 (49%) at least one chronic oral focus of infection, and 219/341 (64%) periodontitis in terms of RAL>3mm. Mean decayed, missing or filled tooth (DMFT) index score was 18.7±7.9 (mean±SD). Of the autologous HSCT recipients, 15/125 (12%) had at least one acute oral focus of infection, 74/125 (59%) had at least one chronic oral focus of infection, and 96/125 (77%) had periodontitis in terms of RAL>3mm. Mean DMFT was 20.1±7.5 (mean±SD) (Table 2).

**Table 1:** Descriptives of the disease and transplant-related factors of the allogeneic and autologous HSCT recipients.

	Allogeneic HSCT recipients			Autologous HSCT recipients		
	Survival <6mo	Survival >6mo	P-value	Survival <6mo	Survival >6mo	P-value
	(n = 47)	(n = 294)		(n = 4)	(n = 121)	
**Male**	26 (55%)	170 (58%)	0,75	3 (75%)	79 (65%)	0,69
**Female**	21 (45%)	124 (42%)		1 (25%)	42 (35%)	
**Age (mean)**	47,6	49,8	0,32	55,5	55,2	0,96
**Smoker**	34,2%	49,1%	0,30	25%	54,5%	**0,01**
**Diagnosis:**						
AML	36,2%	30,6%	0,45	50%	3,3%	**<0,001**
ALL	14,9%	14,3%	0,91			
PCD	8,5%	10,2%	0,72		58,7%	
MH	2,1%	1,9%	0,69		3,3%	
NHL	14,9%	8,5%	0,16	25%	26,4%	0,98
MPN	1,9%	7,5%	0,14			
MDS	14,9%	11,6%	0,52		0,8%	
BMF	4,3%	2,4%	0,46			
Other	2,6%	13%	0,12	25%	7,5%	0,23
**Ablative conditioning**	80,9%	79,3%	0,60	75%	99,1%	0,87
**TBI**	51,1%	42,9%	0,22	0%	2,5%	0,75
**aGVHD:**						
no	48,9%	45,6%	0,67			
grade 1	8,5%	23,8%	**0,02**			
grade 2	10,6%	21,8%	0,08			
grade 3	12,8%	7,1%	0,19			

AML: Acute Myeloid Leukaemia, ALL: Acute Lymphoblastic Leukaemia, PCD: Plasma Cell Dysplasia, MH: Hodging Lymphoma, NHL: Non-Hodging-Lymphoma, MPN: Myeloproliferative Neoplasm, MDS Myelodysplastic Syndrome, BMF: Bone Marrow Failure.

**Table 2:** Allogeneic HSCT recipients with the presence of oral foci of infection and oral diseases according to six months survival.

Baseline presence of:	Survival <6mo post-HSCT (n = 47)	Survival >6mo post-HSCT (n = 294)	P-value*
Acute oral foci of infection (n)	3	22	0,788
Chronic oral foci of infection (n)	25	142	0,553
Periapical pathology (n)	11	74	0,795
Caries profunda (n)	8	31	0,195
Furcation involvement (n)	11	60	0,639
Other foci (n)	8	28	0,120
Number of foci (mean)	1,5	1,1	0,464
Peridontitis (n)	26	193	0,170
RAL (mean)	4,8	5,0	0,411
DMFT (mean)	18,4	18,8	0,760

*binary outcomes: Pearson Chi-squere; Continuous varriables: Mann-Whitney U test or t-test.

Within 6 months post-HSCT, 47/341 (14%) allogeneic and 4/125 (3%) autologous HSCT recipients died. Six allogeneic recipients died within the first 30 days post-HSCT. There were no statistically significant differences between the survivors and non-survivors in terms of transplant-related factors or covariates, including age, sex, smoking, TBI, ablative conditioning or diagnosis. Among allogeneic HSCT recipients, grade IV acute GvHD was associated with death within 6 months post-HSCT (P: <0.001), whereas grade I acute GvHD was associated with survival (P: 0.02). (Table 1)

### Oral foci of infections and patients’ survival

Acute and chronic oral foci of infections, periodontitis and DMFT, were not associated with survival at 6 months post-HSCT among allogeneic HSCT recipients (Table 2). Only 4/125 autologous HSCT recipients died within 6 months post-HSCT, and no statistical analysis was carried out

### Oral foci of infections and hospital treated infectious diseases

There were no associations between any of the oral foci of infections and any infectious diseases (any infection, pneumonia, gut infection, or bacterial/fungal/viral-infection) (Table 3). Among allogeneic HSCT recipients, there was initially a weak association (P = 0.032) between caries profunda and pneumonia. However, after the Bonferroni correction, the association did not reach statistical significance.

**Table 3:** Association of having chronic oral foci of infection with hospital treated infectious diseases 0–6 months post-HSCT.

Baseline presence of:		Allogeneic (n = 341)	Autologous (n = 125)
		with infection (n)	with infection (n)
Any oral foci*	**yes**	101	25
	**no**	98	15
Periodontitis*	**yes**	126	31
	**no**	73	9
Acute oral infection*	**yes**	14	6
	**no**	185	34

* Chi-square: P-value >0.05.

### Oral foci of infections and bacteremia

86/341 (25%) of the allogeneic and 11/125 (9%) of the autologous HSCT recipients had blood culture positive bacteremia. The most common species were *Escherichia coli* and *Staphylococcus epidermidis*. 5/341 (1%) of the allogeneic and 3 (2%) of the autologous HSCT recipients had oral *Streptococcus mitis* group or *Streptococcus salivarius* positive blood cultures. Among autologous HSCT recipients, no associations between chronic oral foci of infections and septic infections were observed. Among autologous HSCT recipients, the presence of “any” chronic oral foci of infection and the presence of “other” foci of infection (root remnants, partially erupted wisdom teeth with pericoronitis, initiated endodontic treatment, or cysts) were initially observed to be associated with bacteremia (P-values: 0.025 and 0.037, respectively). However, after the Bonferroni correction, these associations did not reach statistical significance (Table 4).

**Table 4:** Association of having oral foci of infection with blood culture positive bacteremia within six months post-HSCT.

Baseline presence of:		Allogeneic (n = 341) with bacteremia (n)	Autologous (n = 125) with bacteremia (n)
**Any oral foci**	**yes**	39	10**
	**no**	47	1**
Periodontitis*	**yes**	52	11
	**no**	34	0
Acute oral infection*	**yes**	5	3
	**no**	81	8

* Chi-square: P-value s>0.05.

** P-value: 0,025.

*** P-value: 0,037.

## Discussion

This retrospective study examined the associations of common oral infections with survival and infectious complications in auto- and allogeneic HSCT recipients. The results of the study showed acute and chronic oral foci of infections, DMFT and periodontitis to not be associated with survival or infectious complications including sepsis during the 6-month follow-up period.

The percentage of participants who died within 6 months post-HSCT was 14% among allogeneic and 3% among autologous HSCT recipients. Among the allogeneic HSCT recipients, there were no statistically significant differences between the survivors and non-survivors in terms of age, sex, smoking or transplant-related factors (TBI, ablative conditioning or diagnosis). In line with previous findings, also in this study the severity of acute graft-versus-host disease (aGvHD) was associated with post-transplant survival [19,20]. Mild, grade I aGvHD was associated with survival, whereas severe, grade IV aGvHD was associated with death within 6 months post-HSCT. This finding highlights the complexity of the immunological effects of GvHD. Mild aGvHD may improve survival by decreasing the risk of disease relapse by enhancing graft versus malignancy. On the other hand, severe aGvHD increases mortality by causing organ failure, and predisposes the recipient to life-threatening infections. Within the limits of this study, oral mucositis or presence of oral signs of aGvHD could not be studied. Acute or chronic oral foci of infections could predispose to these disorders of oral mucosa, and further studies are thus warranted.

It is currently unclear which oral infectious diseases should be considered as oral foci of infections and would need to be eliminated prior to high-dose chemotherapy [16]. Additionally, evidence on the benefits of dental treatments before high-dose chemotherapy and HSCT is scarce. Despite the lack of evidence, it is commonly recommended that oral foci of infections should be eliminated, since expected neutropenia during chemotherapy, and especially during the full intensity conditioning regimen pre-HSCT, predisposes patients to a spread of infection [10,14,21]. However, patients diagnosed with acute haematological malignancies, particularly acute myeloid leukaemia and acute lymphoblastic leukaemia, as well as subjects with multiple myeloma and lymphomas, are often treated with high-dose chemotherapy and HSCT abruptly in a salvage setting. Thus, there is often no time pre-HSCT for dental treatments, and they ought not to cause delay to the cancer therapy [10].

In our clinic, all HSCT recipients are referred to a dental examination, which is commonly conducted only a few days pre-HSCT. In this study, acute oral foci of infection were observed in 7% of the allogeneic and 12% of the autologous HSCT recipients, and at least one chronic oral focus of infection in 49% of the allogeneic and 64% of the autologous HSCT recipients. Due to myelo- and immunosuppression, all elective dental treatments are postponed. Thus, all subjects with chronic oral foci of infection proceed into transplantation. However, neither acute nor chronic oral foci of infections were associated with survival at 6 months post-HSCT in this study. Only one previous study concerning the associations of oral foci of infections with survival post-HSCT was found. In line with our results, in that study with 58 allogeneic or autologous HSCT recipients, no statistically significant correlations of oral foci of infections with infections and survival rate post-HSCT were observed [22].

Similarly to the survival, no associations of the examined oral infections with the hospital treated infectious diseases or blood culture positive bacteremia were observed. The most common species in the blood cultures were *Escherichia coli* and *Staphylococcus epidermidis*, which are most often associated with urethritis and venous catheters. Bacterial species that are most likely to originate from the oral cavity were surprisingly uncommon, as only 5 (1%) of the allogeneic and 3 (2%) of the autologous HSCT recipients had oral *Streptococcus mitis* group or *Streptococcus salivarius* positive blood cultures. These results are in line with a previous study with 35 autologous HSCT recipients, of which 6% had acute and 63% chronic oral foci of infection [17]. No statistically significant differences were found between recipients with or without oral foci of infection in terms of duration of neutropenia, fewer or blood culture positive bacteremia. Also in that study, oral-related bacterial species were uncommon, as only 1 subject (3%) was observed with *Streptococcus mitis*. Another recent study among 184 allogeneic HSCT recipients reported 28 (15.2%) cases with viridans streptococcal bacteremia. In a more detailed analysis, 14 strains of *Streptococcus mitis* (7%) were observed. Viridans streptococcal bacteremia was not a risk factor for all-cause mortality up to 60 days following allogeneic HSCT [23].

In the present study, the prevalence of common oral diseases was relatively high at pre-HSCT. 64% of the allogeneic and 77% of the autologous HSCT recipients had periodontitis in terms of RAL>3mm, and mean DMFT scores were 18.7 and 20.1, respectively. In our previous study with 257 healthy Swiss adult subjects, a mean DMFT score of 14.2 was observed [24]. In addition, we have demonstrated hyposalivation to be common in HSCT recipients already pre-HSCT [7]. Hyposalivation related to preceding conditioning and chemotherapies can explain some of the observed oral diseases, the elevated DMFT in particular. Hyposalivation and the lack of defensive functions due to the alternated composition of saliva, that may be related to haematological diseases and their systemic treatment, is to be considered as a causative and in many cases an absolutely unavoidable factor that results in an increasing need of conservative dentistry. An intriguing recent study found patients with acute leukaemia to have poor oral health in terms of caries and periodontitis already prior to any treatments [25]. Poor oral health and high dental treatment needs have been reported also in other studies among HSCT recipients [26]. However, in this study, poor oral health was not associated with survival, as no statistically significant differences in the presence of periodontitis or in DMFT were observed between the survivors and non-survivors. In another previous study, periodontitis was observed in 66% and caries in 72% of the studied 36 HSCT recipients pre-HSCT without any signs or symptoms associated with oral foci of infection while they were immunosuppressed [27].

Similarly to our recent previous study, also in this study periodontitis was determined indirectly by measuring radiological attachment loss from panoramic radiographs [28]. This method was selected to avoid bacteremia and to keep the dental visits as short as possible for the severely ill patients immediately prior to the transplantation. This method can detect only past or present periodontitis and it misses all signs of early disease. On the other hand, some patients that already have been treated for periodontitis may have been diagnosed false positive in terms of RAL>3mm in panoramic radiographs. Thus, the results concerning periodontitis must be treated with caution. However, and despite this relatively coarse method of periodontitis diagnosis, this does not cause bias in the results as both groups were assessed with this RAL-based manner, and no differences between the survivors and non-survivors among the HSCT recipients were observed.

With the retrospective data available for this study, duration of neutropenia, fewer episodes, duration of hospitalization, used prophylaxis and interventions as well as subjective symptoms including quality of life could not be studied. However, oral diseases may cause morbidities like these, leading to excessive and/or prolonged hospital treatments, and being subjectively distressing. Further studies are thus needed to assess the associations of oral diseases with these kinds of less severe problems.

Within the limits of this study, no associations of acute or chronic oral foci of infections, DMFT and RAL with survival or infectious complications including sepsis 6 months after auto- or allogeneic HSCT were observed. Thus, the results support the hypothesis that chronic oral foci of infections could be left untreated and radical dental interventions postponed until after the high-intensity oncologic treatmenst have been completed. Pre-HSCT, the emphasis should be on diagnosis by oral medicine professionals, conservative dental treatments and oral and systemic prophylaxis.
